# Fast Leak-Proof, Intraumbilical, Single-Incision Laparoscopic Ovarian Cystectomy for Huge Ovarian Masses: “Hybrid Cystectomy and Reimplantation” Method

**DOI:** 10.3390/medicina57070680

**Published:** 2021-07-02

**Authors:** Sa Ra Lee

**Affiliations:** Department of Obstetrics and Gynecology, Asan Medical Center, University of Ulsan College of Medicine, 88, Olympic-ro 43-gil, Songpa-gu, Seoul 05505, Korea; leesr@amc.seoul.kr; Tel.: +82-2-3010-3648; Fax: +82-2-3010-3630

**Keywords:** huge ovarian mass, ovarian cystectomy, single-incision laparoscopic surgery

## Abstract

*Background and Objectives*: To introduce a new technique for fast leakage-proof, intraumbilical, single-incision laparoscopic ovarian cystectomy for huge ovarian masses (>10 cm) *Materials and Methods*: Seven consecutive, reproductive-aged women, including three adolescents, with huge ovarian masses (mature cystic teratoma, *n* = 4; endometrioma, *n* = 2; and mucinous cystadenoma, *n* = 1) who underwent transumbilical single-incision ovarian cystectomy with the new “hybrid cystectomy and reimplantation” method were included. The procedure was: (1) trans-umbilical single-incision laparoscopy; (2) inspection of the pelvic cavity and placing the mass in a laparoscopic endo-bag for cystic content leakage prevention; (3) in-bag resection using cold scissors and minimal cauterization of the cystectomy site; (4) in-bag tissue extraction; (5) rapid extracorporeal cystectomy with traction without electrocautery; (6) re-insertion of the retrieved ovarian cortex intracorporeally through the single port, and (7) intracorporeal suture of the retrieved tissue to the in situ ovary. *Results*: The mean patient age was 24.71 ± 6.56 (range 17–37) years and the mean maximal diameter of the masses was 17.71 ± 2.86 (range 13–22) cm. There was no case of unintended intracorporeal cyst rupture and no need for copious irrigation for washing and suctioning the leaked mass content. The mean total operating time was 76.42 ± 6.39 (range 65–85) min, the total volume of saline used for irrigation was 814.28 ± 331.35 (range 500–1500) mL, and the estimated blood loss was 107.14 ± 47.72 (range 50–200) mL. There were no perioperative complications. All patients except the two endometriosis patients had regular, normal menstruation. *Conclusions*: Our preliminary findings were encouraging in terms of the safety and efficiency of the new method. Future trials need to elucidate the benefits of this method in terms of fertility preservation.

## 1. Introduction

Ovarian neoplasms in reproductive-aged women are a common gynecologic problem that requires surgical interventions. Ovarian neoplasms can be classified as cystic, solid, or complex (a cystic mass with solid components or septations) according to the structure of the mass, as well as benign, borderline malignant, or malignant according to the malignancy potential. Benign ovarian tumors account for 90% of all ovarian tumors [1]. They are usually unilateral, cystic, movable, and smooth with minimal or no ascites; additionally, they demonstrate slow growth and are frequently found in younger patients [2].

Ovarian cystectomy for benign neoplasms instead of oophorectomy is mandatory for ovarian function preservation [3,4,5]. Even in ovarian borderline malignancies, cystectomy is often chosen for fertility preservation. In reproductive-aged women without plans for pregnancy, preservation of normal ovarian tissue and function during surgery is also important to prevent early surgical menopause or deterioration of postoperative ovarian function. Surgeons aim to preserve as much normal ovarian tissue as possible during ovarian cystectomy in reproductive-aged women. Damage to the ovarian vascularity and removal of a considerable amount of ovarian tissue can result in a reduction of the ovarian reserve [6,7]. Bipolar electrocoagulation during ovarian cystectomy is also accompanied by a low ovarian reserve, which is indicated by lower serum anti-Mullerian hormone (AMH) levels and a lower follicle count [8,9]. Therefore, choosing a surgical technique with minimal use of electrocauterization is important during ovarian cystectomy to preserve the ovarian reserve. In terms of surgical methods, minimally invasive surgery (MIS) is preferred in most ovarian cystectomy cases due to its advantages of minimal scarring, less postoperative pain, and fast postoperative recovery [4,6,10]. Furthermore, following recent advances in laparoscopic instruments, including energy devices and diverse multi-channel single-port platforms, single-incision laparoscopy has become the preferred surgical option for ovarian cystectomy to minimize postoperative scarring further [11].

However, huge ovarian masses (>10 cm) with a solid and complex nature can often be a challenge in cystectomy with MIS or single-incision laparoscopy because of the risk of cyst rupture and a non-aspirable mass content [11]. Cyst rupture can be associated with chemical peritonitis in mature cystic teratoma (MCT) cases or endometrioma and pseudomyxoma peritonei in mucinous tumor cases [12]. Even though the correlation between cyst rupture and these complications is controversial, the total operative time (OT) can be increased due to the need for additional surgical time for copious intrapelvic irrigation. Sometimes, intracorporeal identification of the cleavage plane and dissection of the ovarian cortical tumor plane are very difficult because of the dense adhesion of the ovarian mass to the normal ovary. In these cases, some of the normal ovarian cortex may be unintentionally resected because of its close association with the ovarian mass during cystectomy and this can affect the ovarian reserve. Therefore, some gynecologists prefer open laparotomy for such challenging cases for better preservation of normal ovarian tissue and to reduce the risk of intraoperative cyst rupture as well as the use of electrocautery or vessel-sealing energy devices in MIS. In addition, the disadvantages of MIS cystectomy in such challenging cases, compared with open laparotomy, include a longer OT in cases of cystic content leakage into the pelvic cavity, the requirement of copious irrigation to prevent postoperative adhesion or chemical peritonitis, and the limited angle and traction power due to the relatively small working parts of laparoscopic instruments.

To overcome these obstacles, some authors have suggested new MIS methods for huge ovarian masses, such as in-bag cystectomy, leakage-proof extracorporeal drainage, and extracorporeal cystectomy [13,14,15,16]. However, these methods have limited scope in some cases, such as when the pelvic masses have solid or a non-aspirable cystic content; when there is a multicystic ovarian mass with thick septations; or the masses are not large enough to access at the level of the umbilicus incision made for the multi-channel single-port platforms [11]. Furthermore, some methods describe oophorectomy, and not cystectomy, even in cases where preservation of ovarian function and fertility is desired in young women [17,18].

Therefore, this study describes a novel, fast, leakage-proof, ovarian tissue-preserving surgical technique of trans-umbilical single-incision ovarian cystectomy applicable for huge ovarian masses regardless of mass content or accessibility.

## 2. Materials and Methods

### 2.1. Study Design and Patients

Seven consecutive, reproductive-aged women, including three adolescents, with huge ovarian masses (>10 cm) who underwent trans-umbilical single-incision ovarian cystectomy with the new “hybrid cystectomy and reimplantation” method between April 2020, and February 2021, were included in this preliminary study. This method was initially developed in April 2020 and was performed by a single surgeon (Dr. L.S.R.), an experienced MIS surgeon who has completed over 3500 laparoscopic surgeries and 1030 robotic surgeries.

We obtained the following data from each patient’s medical charts: age; body mass index (BMI); detailed gynecologic, medical, and surgical histories; and the size of the ovarian mass (maximal horizontal and vertical diameter of the mass in centimeters) measured using preoperative ultrasonography or computed tomography. AMH levels were measured both preoperatively and 3 months postoperatively and then only when it was necessary to be measured during usual clinical practice. Patients with an adnexal mass with laboratory or imaging findings suggesting malignancy were excluded from this study. The median serum cancer antigen 125 (CA 125) level was 22.1 ± 11.40 (range 17–50) U/mL.

The following surgery-related data were recorded: total OT from skin incision to closure and total volume of normal saline used for irrigation of the pelvic cavity intraoperatively. Perioperative outcome data were collected, such as the estimated blood loss, intra- or postoperative adverse events, length of hospital stay, and complications related to this cystectomy method.

### 2.2. Surgical Procedures

**Figure 1:**
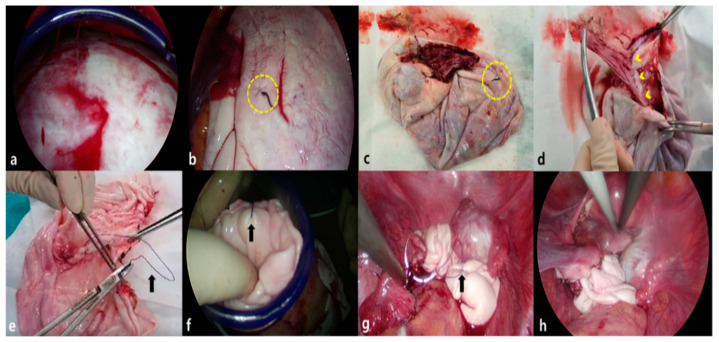
The hybrid cystectomy and reimplantation method (case 2). (**a**) The huge ovarian mass is directly visible through a single laparoscopic port inserted in the umbilicus. (**b**) Laparoscopic view; the decompressed ovarian cyst after suctioning the cystic fluid out using the OSCHNER trocar. The puncture site is sealed with the purse-string suture and tied with black silk (dotted circle) after suctioning out the fluid contents. (**c**) Extracted ovarian cystectomy specimen with the solid contents, the initial purse-string suture site is noted (dotted circle). (**d**) Extracorporeal cystectomy was performed very easily using long Kelly clamps on the back table without limitation of the range of motion as occurs in intracorporeal cystectomy. The dissection plane is obvious (tumor-normal ovarian cortex plane, arrowheads). (**e**) The retrieved ovarian cortex piece is rinsed in normal saline and is approximated to make an ovarian shape with barbed suture material, 1-0 Quill™ SRS (arrow) in a continuous running manner. (**f**) The retrieved ovarian cortex is placed back into the pelvic cavity through the single laparoscopic port with the suture material (arrow) that was used during procedure. (**g**) Re-implantation of the retrieved ovarian cortex into the cystectomy site of the in situ ovary using the suture material (arrow) inserted into the pelvic cavity during procedure. (**h**) Completion of the cystectomy and a large volume of the ovary remains in situ.

**Figure 2:**
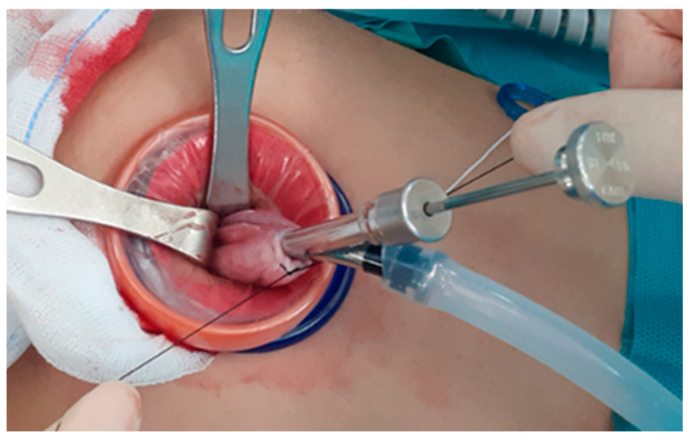
In cases of very large ovarian masses that could not be placed even in an extra-large endo-bag, the size of the mass was decreased using a technique of placing a purse-string suture on the surface of the ovary and inserting the OSCHNER trocar into the mass through the circle of the purse-string sutures to drain the cystic contents. When there is nothing left to be drained, the suture is tied while simultaneously drawing the OSCHNER trocar out of the cyst to seal the trocar insertion site to prevent spillage of the cystic contents.

A 2.0–2.5 cm vertical incision is made at the umbilicus using the open-Hassel technique and a multi-channel single-port trocar, Gloveport A-Type (Nelis Meditech Inframed, Ojeong-gu, Korea), is inserted. A trans-umbilical single-incision laparoscopic ovarian cystectomy is performed using this new hybrid cystectomy and reimplantation method (Figure 1) as follows: (i) A 2.0–2.5 cm intra-umbilical incision is made. (ii) A multi-channel single-port trocar is inserted and we position the patient in a steep Trendelenburg position for moving the ovarian mass in the cephalic direction toward the level of the umbilicus. (iii) The huge ovarian cyst is placed in a laparoscopic endo-bag to prevent spillage of the cystic contents during cystectomy (intracorporeal in-container cystectomy). In cases of adhesions noted around ovarian cysts, especially in cases of endometrioma, adhesiolysis with monopolar scissors is used to free the ovarian mass before putting it in the endo-bag. However, in cases of very large ovarian masses that could not fit in even an extra-large endo-bag, the size of the mass is decreased using a technique reported previously [11,14,18,19]; thereafter, the reduced ovarian mass is placed in the endo-bag. This technique includes placing a purse-string suture on the surface of the ovary and inserting the OSCHNER trocar into the mass through the circle of the purse-string sutures to drain the cystic contents as described in previous reports [11,18,19,20]. When there is nothing left to be drained (such as in case of blockage of the suction system due to thick fat, hair, scalp, or bone-in a teratoma or thick septum in a multiseptated mucinous cyst), the suture is tied while simultaneously withdrawing the OSCHNER trocar out of the cyst to seal the trocar insertion site to prevent the spillage the cystic contents (Figure 2). (iv) In-bag resection of the mass is performed using monopolar scissors with minimal use of electrocautery. (v) The resected ovarian cyst is exteriorized without extension of the surgical wound. (vi) Subsequently, extracorporeal ovarian cystectomy is performed. Dissection of the ovarian cortical- tumor plane is performed extracorporeally on the back-table using Kelly forceps or Allis forceps without the use of electrocautery. (vii) The retrieved ovarian cortex is washed in normal saline. (viii) Extracorporeal continuous running suturing of the edge of the retrieved ovary is performed to make an oval shape to facilitate intracorporeal suturing. (ix) Re-insertion of the retrieved ovarian cortex intracorporeally is performed through the single port and with the needle on the thread in situ. (x) Finally, intracorporeal suturing of the re-inserted ovarian tissue to the in situ ovary (ovarian cystectomy site) is performed.

All surgeries were performed under general anesthesia, and all patients received standard operative care. Conventional laparoscopic instruments including a 10 mm rigid camera, monopolar scissors, grasper forceps (ENDOPATH^®^ grasper, 5 mm, Ethicon, Cincinnati, OH, USA), dissector forceps (ENDOPATH^®^ bipolar forceps, 5 mm, Ethicon), and an intracorporeal needle holder (Durogrip TC needle holder, straight, 5 mm, Aesculap) were used. The Endo-bag (Sejong Medical, Paju, Gyeonggi-do, Korea) or Endo-pouch (Nelis Meditech Inframed) were used in all cases. Intracorporeal ovarian suturing was performed using absorbable barbed suture materials (2-0 Monofix PDO; Hanmi, Daejeon, Korea) or unidirectional 1-0 or 2-0 sutures (Quill™ SRS; Angiotech Pharmaceuticals, Inc., Vancouver, BC, Canada). Intraoperative biopsy for evaluation of the malignancy was not performed because this method was only used for patients with an adnexal mass without any signs of malignancy during the preoperative evaluation of laboratory and imaging studies.

### 2.3. Statistical Analysis

Data are expressed as mean ± standard deviation unless stated otherwise. All statistical analyses were performed using R (R Foundation for Statistical Computing, Vienna, Austria) [21].

## 3. Results

### 3.1. Patient Baseline Characteristics

**Figure 3:**
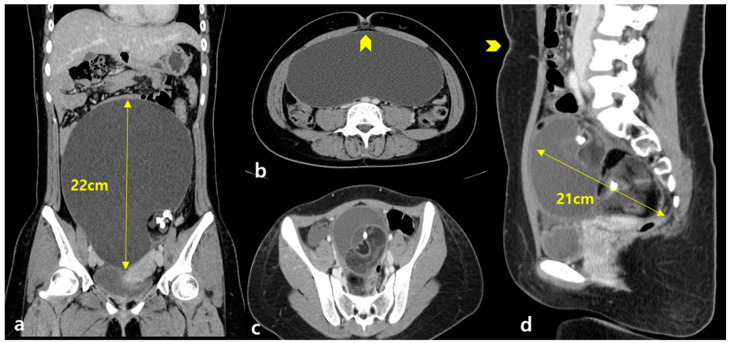
(**a**) A 17-year-old woman with a huge abdominal mass (Case 1). A coronal view on computed tomography (CT) revealed calcification within a 22 × 20 cm ovarian mass with low signal intensity on T2 weighted imaging (T2WI), suggesting a mature cystic teratoma. (**b**) An axial view, umbilicus (arrowhead) (**c**) of an 18-year-old woman with bilateral huge abdominal masses (Case 2). An axial view revealed calcification within a 17 × 15 cm ovarian multi-cystic mass and another 8 × 7 cm mass with low signal intensity on T2WI, suggesting a mature cystic teratoma. (**d**) A sagittal view, umbilicus (arrowhead).

Seven reproductive-aged patients with huge benign ovarian cysts (>10 cm) underwent single-incision laparoscopic ovarian cystectomy (MCT, *n* = 4; endometrioma, *n* = 2; and mucinous cystadenoma, *n* = 1) (Table 1). The mean patient age was 24.71 ± 6.56 (range 17–37) years, and all patients were of reproductive age with nulligravida. The mean maximal diameter of the mass was 17.71 ± 2.86 (range 13–22) cm, one patient (Case 2) had a bilateral ovarian mass, and the others had a unilateral ovarian mass (Figure 3). None of them had previously undergone ovarian surgery or chemotherapy, which could affect their ovarian reserve. None of them were smokers (Table 1).

### 3.2. Perioperative Outcomes

Surgeries for all cases were completed using a trans-umbilical single-incision method with no visible leakage or conversion to laparotomy or multi-port laparoscopy. There was no case of unintended intracorporeal cyst rupture and no need for copious irrigation for washing and suctioning of the leaked mass content. The mean total OT was 76.42 ± 6.39 (range 65–85) min, the total volume of saline used for irrigation was 814.28 ± 331.35 (range 500–1500) mL, and the EBL was 107.14 ± 47.72 (range 50–200) mL. The time taken for extracorporeal cystectomy at the back-table was 5–10 min.

Preoperative and 3-month postoperative serum levels of AMH were available in seven and five cases, respectively. The mean preoperative level was 3.26 ± 0.88 (range 2.1–4.5) ng/mL. Two patients (Cases 5 and 6) underwent postoperative medical treatments, including gonadotropin-releasing hormone agonist (leuprolide acetate, 3.75 mg subcutaneously, monthly for 3 months), for the prevention of endometriosis recurrence and their postoperative serum AMH levels could not be accurately measured.

**Figure 4:**
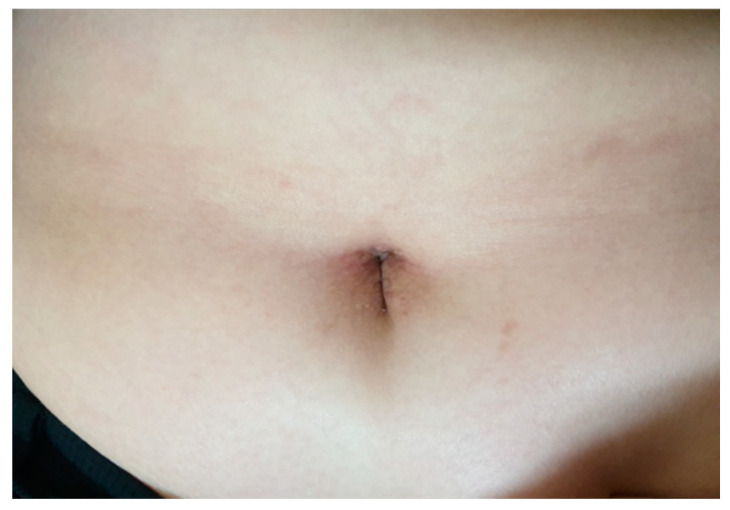
Abdominal wound scar at the postoperative 2-week follow-up (Case 1). No visible scar with only slight discoloration around the umbilicus.

All patients were discharged on the 2nd postoperative day and no perioperative complications related to cystectomy were observed. The abdominal wound at postoperative 2-week follow-up (Case 1) revealed no visible scarring with only slight discoloration around the umbilicus (Figure 4). All patients except the two endometriosis patients (Case 5 and 6) had regular, normal menstruation at the postoperative 3-month follow-up.

## 4. Discussion

The surgical approach for large ovarian cysts in young women who desire fertility preservation remains a challenge. We must not only consider maximum preservation of the normal ovary with minimal injury to the vasculature and damage following electrocauterization [1,6,7,8,9] but also minimize the leakage of the cystic contents into the pelvic cavity to decrease the need for copious irrigation [11,13,15,17,18], which increases the total OT, along with making minimal scarring for cosmetic purposes [19,20,22].

In this study, we introduce a new hybrid cystectomy and reimplantation method for fast, leakage-proof, single-incision ovarian cystectomy for huge ovarian masses. With this method, we can achieve: a decrease in the amount of normal ovarian tissue attached to the ovarian mass inadvertently being discarded during cystectomy; completely prevent leakage of the content of the ovarian mass into the pelvic cavity and thus reduce the risk of chemical peritonitis; decrease the total OT by eliminating the time required for suction and copious irrigation after leakage of the contents into the pelvic cavity and eliminate the time necessary for intracorporeal cystectomy using the limited power and angles of the standard laparoscopic instruments, and reducing postoperative scarring by enabling surgery completion using a trans-umbilical single-incision method even in cases of huge ovarian masses without signs of malignancy in the preoperative laboratory or imaging studies.

### 4.1. Previous Reports on Surgical Techniques for Huge Ovarian Masses

A randomized controlled trial, published in 2007, compared 4 port laparoscopy and laparoscopically-guided mini-laparotomy (3–7 cm transverse skin incision 2–4 cm above the symphysis pubis) for huge ovarian masses (median diameters of 8.2 and 8.4 cm, respectively) [22]. The authors concluded that the laparoscopically-guided mini-laparotomy could result in reduced intraperitoneal leakage compared with laparoscopy; therefore, it should be preferred over traditional laparotomy even for large ovarian cysts [22]. Chong et al. [20] also reported a single port-assisted extracorporeal cystectomy in 25 patients and compared the surgical outcomes, complications, and leakage rate with those of conventional laparoscopy and laparotomy for large ovarian masses (>8 cm) [20]. They concluded that these three surgical approaches had similar surgical outcomes; therefore, single-port surgery can be an alternative to conventional laparoscopy and laparotomy [20]. The mean diameter of the ovarian masses in these groups was 11.4, 9.7, and 12.0 cm, which are large but not huge compared with the measurements in our study and Yi’s report [11]. They performed extracorporeal suturing using conventional laparotomy instruments at the umbilical level, which is in contrast to our intracorporeal suturing of the ovary in situ. Attached ovarian masses cannot be completely exposed through the port placed within the umbilicus in cases of masses that are not large enough to reach the umbilicus; therefore, Weng et al. made a 4 cm skin incision 3 cm above the symphysis pubis and not within the umbilicus [17].

However, our proposed method can be applied to ovarian masses irrespective of whether they can be exposed through the umbilical port or not. Extracorporeal cystectomy or oophorectomy for huge ovarian masses has been previously reported [11,17,18,19,20] but these reports were based on mini-laparotomy [17,18] or conventional laparoscopy, which needs an extension of the 5 mm or 10 mm incision of the trocar site for the removal of huge masses [11,19,20,22]. Recently, a novel technique was introduced for performing intraperitoneal ultrasound scans by culdotomy to guide precise laparoscopic cystectomy for complex ovarian tumors, and in this method, the tumor can be extracted within an endo bag, through the posterior colpotomy [23].

### 4.2. Previous Reports on Leakage-Proof Methods for Huge Ovarian Masses

In terms of leakage-proof methods for resecting huge ovarian masses, Shozu et al. first reported a leakage-proof puncture method for 30 patients with large ovarian masses (range, 5–27 cm; mean, 15 cm) using a polyethylene bag placed directly over the ovarian cyst using cyanoacrylate adhesive and a 3–5 cm mini-laparotomy approach [15]. Weng et al. reported a similar technique using a polyurethane membrane instead of the adhesive and a self-retraining wound retractor using a mini-laparotomy approach in 20 patients with large ovarian tumors (range, 10–26 cm; mean, 15 cm) [17]. A 4 cm skin incision was placed 3 cm above the symphysis pubis, and salpingo-oophorectomy was performed extracorporeally. Overall, success was observed in 18 of the 20 cases; the exceptions were a patient with obesity and one with severe adhesions [17]. Recently, another article reported a leakage-proof extracorporeal drainage technique performed in 17 pediatric and adolescent patients with ovarian neoplasms over a period of 8 years; however, this technique involved a low Pfannenstiel incision of 3–7 cm [18]. Furthermore, ovarian cystectomy was performed only in 29.4% of the patients, and the others were oophorectomy cases, even in the 71% of the cases that were 11 young prepubertal girls with MCT [18]. In the current study, none of the patients had postoperative complications, including chemical peritonitis or hypothermia, which can be associated with the long OT, with a mean total OT under 80 min and the total volume of saline used for irrigation was less than 850 mL.

### 4.3. Previous Reports on Single-Incision Laparoscopy for Huge Ovarian Masses

Extracorporeal cystectomy using single-incision laparoscopy has been previously reported [11,19,20,24]. Yi reported three cases of MIS ovarian cystectomy for huge ovarian cysts (13.9, 21.6, and 34.0 cm) using the laparoscopic extracorporeal approach, which is similar to our method. However, we did not use electrocautery except to control bleeding at the cystectomy site, and our method can also be applied to all kinds of ovarian masses regardless of their content, contrary to this method [11]. To prevent leakage of the cystic contents during aspiration, Yi et al. inserted pieces of a wet surgical gauze around the inner edges of the wound retractor [11]. Although they did not use electrocoagulation within the abdominal cavity, the handswitch Bovie coagulator was used in the extracorporeal cystectomy in contrast to our method, in which we used only Kelly or Allis clamps without electrocautery [11]. The relative risk of leakage is high in single-incision laparoscopy for large adnexal masses (>7 cm) if the entire procedure is performed only intracorporeally without extracorporeal cystectomy [19]. Yi et al. preferred a hand-assisted single-port laparoscopic surgery to pure single-port laparoscopic surgery that involves only an intracorporeal procedure [11]. Although they described successful hand-assisted single-port laparoscopic surgery in masses with a mean maximum diameter of 10.9 (range 6.5–26.8) cm, adnexal masses of <10 cm occasionally could not reach the umbilicus even in the steep Trendelenburg position, and extracorporeal cystectomy through a single-port intraumbilical procedure is not possible in our experience. Song et al. reported a single-port laparoscopic ovarian cystectomy with a technique similar to ours in 17 patients with extremely large ovarian cysts (≥15 cm) [24]; however, our technique enables single-port laparoscopic ovarian cystectomy to be performed even for large adnexal masses that are large enough to reach the umbilicus.

Extracorporeal traction of the tissue using only long Kelly or Allis clamps during ovarian cystectomy, as in our method, is considerably faster and easier than that with conventional laparoscopic atraumatic forceps with small jaws intracorporeally; additionally, we can also easily observe the cleavage plane (tumor-normal ovarian cortex plane). These procedures took <5 min in all cases. In terms of the OT from skin incision to skin closure for single-incision laparoscopic ovarian cystectomy for large ovarian masses, the median OT was 79.8 min (39–155), including 2 cases of adnexectomy and 3 cases of staging for borderline/malignant tumor [24], 69.3 min in 25 cases [20], and 93 min in 3 cases [11], which are similar to our results (85 min in 7 cases).

The difference in our technique compared with previous reports involving extracorporeal cleavage and suturing during ovarian cystectomy is that previous studies used electrocautery for extracorporeal cystectomy [11,20,24] and suturing was performed extracorporeally; however, we do not use electrocautery during extracorporeal cystectomy and suturing was performed intracorporeally during reimplantation of the retrieved ovarian cortex. Therefore, our method can be applied to all ovarian masses even when the mass is not sufficiently large to reach the umbilicus (usually ovarian masses 10–15 cm in size).

Although our method required a shorter time for complete detachment of the normal ovarian tissue attached to the ovarian mass, the process of exteriorization, cystectomy, and reimplantation may affect the ovarian reserve. When we consider the process involved in cryopreserved ovarian cortex transplantation, which is usually performed in the oncofertility field, the ovarian cortex is usually placed in the ovarian fossa or retroperitoneal space, and the resumption of a natural menstrual cycle and spontaneous pregnancy have been reported [13,25,26,27,28]. Furthermore, the ovarian tissue color before and after reimplantation appeared normal. However, to draw any conclusions about the effect of our method on fertility preservation, a randomized controlled trial is mandatory.

### 4.4. Strengths and Limitations

This study had several strengths. First, to the best of our knowledge, this is the first study to report a new hybrid cystectomy and reimplantation method. None of the aforementioned reports have described cystectomy at the back-table without electrocauterization or the use of intracorporeal sutures for reimplantation of the retrieved ovarian cortex. Second, nearly all of the ovarian cortex can be preserved with this procedure, except the electrocauterized ovarian tissue at the time of the first step of intracorporeal cystectomy. Third, single-incision cystectomy can be performed within a short OT. Fourth, this method can be applied to nearly all huge ovarian cysts regardless of mass size or non-aspirable contents. Finally, none of the patients required additional skin incisions or mini-laparotomy, and only one intra-umbilical incision is needed for our technique.

Nevertheless, this study also had some limitations. First, this was a preliminary report and not a multi-center randomized controlled trial. Second, it included a small number of patients and the short follow-up data precluded us from deriving any conclusions about the long-term efficacy and safety of this method. Third, we only evaluated the ovarian reserve based on serum AMH levels without using any other markers, such as the antral follicle count on sonography. Fourth, factors that can affect the ovarian reserve, such as polycystic ovary syndrome, were not adjusted for in the analyses. Finally, a single surgeon familiar with the intracorporeal sutures using single-incision laparoscopy performed all the surgeries; therefore, we cannot exclude the possibility of interpersonal variations when applying this method. In addition, this surgical method only can be applied in cases of ovarian masses without signs of malignancy in the preoperative laboratory or imaging studies.

## 5. Conclusions

In conclusion, our preliminary findings were encouraging in terms of the safety and efficiency of the new method. Future trials are necessary to elucidate the benefits of this method in terms of fertility preservation.

## Figures and Tables

**Table 1:** Patient baseline characteristics and perioperative outcomes.

Case	Age (Years)	Mass Size (cm^2^)	Total OT (min)	Irrigation Volume (mL)	EBL (mL)	Final Pathologic Diagnosis	Pre-Op AMH (ng/mL)	Post-Op AMH (ng/mL)
Case 1	17	22 × 20	65	600	70	MCT	4.1	3.8
Case 2	18	17 × 15, 8 × 7	75	500	100	MCT	3.8	3.2
Case 3	24	20 × 18	80	900	80	MCT+mucinous cystadenoma	2.5	2.1
Case 4	23	16 × 12	85	500	50	MCT	4.5	4.0
Case 5	37	13 × 11	80	1500	150	EMS	2.3	N.A.
Case 6	31	16 × 14	70	1000	200	EMS	2.1	N.A.
Case 7	23	20 × 20	80	700	100	mucinous cystadenoma	3.5	3.2

SD, standard deviation; BMI, body mass index; OT, operative time; EBL, estimated blood loss; AMH, anti-Mullerian hormone; MCT, mature cystic teratoma; EMS, endometriosis. N.A., not available

## Data Availability

The Excel data used to support the findings of this study were supplied by Sa Ra Lee under license, and requests for access to these data should be made to S.R.L. leesr@amc.seoul.kr.
